# Embedded Upconversion Nanoparticles in Magnetic Molecularly Imprinted Polymers for Photodynamic Therapy of Hepatocellular Carcinoma

**DOI:** 10.3390/biomedicines9121923

**Published:** 2021-12-15

**Authors:** Cheng-Chih Lin, Hung-Yin Lin, James L. Thomas, Jia-Xin Yu, Chien-Yu Lin, Yu-Hua Chang, Mei-Hwa Lee, Tzong-Liu Wang

**Affiliations:** 1 Division of Pulmonary Medicine, Department of Internal Medicine, Armed-Forces Zuoying General Hospital, Kaohsiung 81342, Taiwan; chengchihlinmike@gmail.com; 2 Department of Electrical Engineering, National University of Kaohsiung, Kaohsiung 81148, Taiwan; 3 Department of Chemical and Materials Engineering, National University of Kaohsiung, Kaohsiung 81148, Taiwan; linhy@ntu.edu.tw (H.-Y.L.); a0982788216@gmail.com (J.-X.Y.); oaqoaq557788@gmail.com (C.-Y.L.); changyuhua880115@gmail.com (Y.-H.C.); 4 Department of Physics and Astronomy, University of New Mexico, Albuquerque, NM 87131, USA; jthomas@unm.edu; 5 Department of Materials Science and Engineering, I-Shou University, Kaohsiung 84001, Taiwan

**Keywords:** upconversion nanoparticles, peptide-imprinted polymer, composite particles, photodynamic therapy

## Abstract

In this work, high-temperature pyrolysis was used to prepare both the core and shell of lantha-nide-doped UCNPs with lithium yttrium tetrafluoride (LiYF_4_) to enhance the green luminescence. Merocyanine 540 (MC540)-grafted magnetic nanoparticles were incorporated in the PD-L1 pep-tide-imprinted poly(ethylene-*co*-vinyl alcohol) particles, which were formed by precipitation in a non-solvent. UCNPs in the non-solvent bath were thus entrapped in the imprinted particles to generate composite nanoparticles for the targeting and photodynamic therapy of PD-L1 in tumor cells. Finally, the in vitro cytotoxicity of the nanoparticles in HepG2 human liver cancer cells was evaluated with the continuous administration of MC540/MNPs@MIPs/UCNPs under irradiation by an NIR laser. To understand the delivery of the UCNP-embedded molecularly imprinted pol-ymers, the intrinsic and extrinsic pathways were also investigated.

## 1. Introduction

Upconversion nanoparticles (UCNPs) show great promise for photodynamic therapy, as they permit the use of deeply penetrating near-infrared (NIR) light to activate photosensitizer (PS) molecules that require the energy of a visible photon to produce singlet oxygen. Liang and Liu have reviewed the non-invasive treatment of cancers using photodynamic therapy (PDT) and upconversion nanoparticles [1]. UCNPs can also be employed in imaging-guided combined photothermal and photodynamic therapy with surface modification by a targeting protein [2]. A recent review of the use of lanthanide-doped UCNPs for drug and gene delivery found that these UCNPs could emit visible light and even UV when irradiated by NIR; the resulting photochemical reactions could deliver drugs/genes more efficiently and safely than could be achieved with conventional free drugs [3]. Our earlier work demonstrated the synthesis of lanthanide-doped core/shell UCNPs of LiYF_4_:Yb^3+^/Er^3+^/Ho^3+^/Tm^3+^@LiYF_4_:Yb^3+^, and showed that the photoluminescence (PL) spectra of these tetragonal-phase nanoparticles could be tuned by varying the doping ratio of the lanthanide ions (Er^3+^, Ho^3+^, Tm^3+^). Photoluminescence intensities were increased by the addition of a shell layer [4]. UCNPs have been simultaneously loaded with chlorin e6, a photosensitizer, and imiquimod (R837), which is a toll-like receptor 7 agonist, for promoting strong antitumor immune responses [5]. Dual stimuli-responsive block copolymers have been used to encapsulate the UCNPs of LiYF_4_:Yb^3+^_0.25_,Tm^3+^_0.01_@LiYF_4_:Yb^3+^_0.2_ and Nile red, with the trigger of controlled release by NIR [6].

Molecularly imprinted polymers (MIPs) were initially developed about half a century ago, and are used in biomedical delivery [7,8,9,10] and sensing [11,12,13]. MIPs have been combined with UCNPs to make fluorescent sensors, for example, for the specific recognition of sterigmatocystin [14]. MIPs combined with silica-coated UCNPs have been synthesized for the detection of ochratoxin A in corn, rice, and feed [15]. Our previous work showed that the in situ polymerization of MIP nanocomposites with UCNPs could be used for the recognition of cysteine, albumin, and hemoglobin [16]. Epitope imprinting has recently been used for larger targets, especially proteins and cells; a thorough review, with a focus on applications of the imprinted polymers in imaging, drug delivery, diagnostics, and tissue engineering, has been published by Piletsky et al. [17]. Recently, dual-template MIPs (doxorubicin and an epitope of CD59 protein), with fluorescent silica nanoparticles at the core and encapsulating gadolinium-doped silicon quantum dots and photosensitizers (Ce6), were designed for cellular targeting, treatment, and imaging [18]. Similarly, fluorescent calcium peroxide has been encapsulated as an imaging probe and a source of H_2_O_2_ [19]; in that work, the exposed peptide in the CD47 extracellular region was used as a template, with copper acrylate as one of the functional monomers and N,N‘-bisacrylylcystamine as a crosslinker. Our recent work demonstrated that photosensitizers could be immobilized on the surface of magnetic nanoparticles, which could then be coated with MIPs imprinted with one peptide sequence from the programmed death-ligand 1 protein (PD-L1), for use in combined photodynamic therapy and immunotherapy [20].

In the present work, lanthanide-doped upconversion core/shell nanoparticles were synthesized and then embedded into MIPs formed by phase transfer imprinting of the PD-L1 peptide, to produce MC540/MNPs@MIPs/UCNP composite imprinted particles. All composite particles were then characterized by transmission electron microscopy (SEM), X-ray diffraction (XRD), Fourier-transform infrared spectroscopy (FTIR), and photoluminescence (PL). Finally, the in vitro cytotoxicity of a combination of PDT and immunotherapy with UCNP composite particles was studied in HepG2 cells, as well as the activation of genes of the apoptosis pathway.

## 2. Materials and Methods

### 2.1. Reagents

Yttrium (III) oxide (Y_2_O_3_ 99.99%), ytterbium (III) oxide (Yb_2_O_3_, 99.99%), and holmium (III) oxide (Ho_2_O_3_ 99.99%), trifluoroacetic acid (TFA, 99.99%), oleic acid (90%), and 1-octadecene (90%) were from Alfa Aesar (Ward Hill, MA, USA). The peptide EDLKVQHSSYRQRA from the sequence of programmed death-ligand 1 (PD-L1) was ordered from Yao-Hong Biotechnology Inc. (HPLC-grade, New Taipei City, Taiwan). Lithium carbonate (Li_2_CO_3_, 98%), poly(ethylene-*co-*vinyl alcohol), EVAL, with ethylene 38 mol%, 3-triethoxysilylpropylamine (APTES), merocyanine 540 (MC540) and RT-PCR primers (Table 1), were from Sigma-Aldrich Co. (St. Louis, MO, USA). Dimethyl sulfoxide (DMSO), from Panreac (Barcelona, Spain), was used as the solvent to dissolve EVAL polymer particles at a concentration of 1 wt%. Absolute ethyl alcohol and 95% ethanol were from J. T. Baker (ACS grade, NJ, USA) and *ECHO* Chemical Co. (Miaoli, Taiwan), respectively.

### 2.2. Synthesis of Core/Shell UCNPs

The synthesis of the core/shell LiYF_4_:Yb^3+^_0.25_/Ho^3+^_0.01_@LiYF_4_:Yb^3+^_0.2_ was modified from our previous work [16]; briefly, (1) Li_2_CO_3_ (1.48 mmol), Y_2_O_3_ (0.74 mmol), Yb_2_O_3_ (0.2 mmol), and Ho_2_O_3_ (0.02 mmol) were dissolved in 50% trifluoroacetic acid 10 mL at 90 °C in a three-necked flask for 30 min. (2) This solution was evaporated to dryness under an argon gas purge, and then equal volumes of oleic acid and 1-octadecene 15 mL were added, and heated to 120 °C at 2 °C/min in 30 min. (3) This light-yellow solution was further heated to 300 °C at 30 °C/min under argon gas, which was kept at 300 °C under vigorous stirring for another 1 h. (4) This mixture was cooled to room temperature (ca. 25 °C) and precipitated with ethanol. (5) The solid was collected by centrifugation at 8000 rpm for 10 min, and then dispersed/reprecipitated from ethanol twice to obtain the oleate-capped LiYF_4_:Yb_0.25_^3+^/Ho_0.01_^3+^ core nanoparticles. For the coating shell layer, a similar approach was taken to the synthesis of the core particles, but without the addition of Ho_2_O_3_ in step (1), and the core LiYF_4_:Yb^3+^_0.25_/Ho^3+^_0.01_ UCNPs was added to the equal volumes of oleic acid and 1-octadecene in step (2) just before heating to 120 °C at 2 °C/min in 30 min.

### 2.3. Formation of Peptide-Imprinted Composite Nanoparticles

The synthesis of MC540-grafted MNPs in the UCNP-embedded peptide-imprinted composite nanoparticles included the following steps: (1) The magnetic nanoparticles were synthesized by co-precipitation of a mixture of iron (III) chloride 6-hydrate and iron (II) sulfate 7-hydrate by sodium hydroxide, and then were repeatedly washed while adsorbed on a magnetic plate [21]. (2) Twenty milligrams of MNPs was grafted with 180 μL APTES and 50 mL 95% ethanol, and this mixture was stirred in a water bath at 90 °C for 2 h. The ATPES-grafted MNPs were rinsed three time with 95% ethanol and kept in 10 mL 95% ethanol. (3) Five milliliters of 0.5 mg/mL MC540 was added to 2 mg of ATPES-grafted MNPs and reacted for 1 h at 60 °C. (4) The peptide was dissolved in DMSO at a concentration of 100 μg/mL, and 250 μL EVAL/DMSO solution was added into the same volume of peptide solution to form a clear EVAL solution, and 10 mg of the MC540/MNPs were then added. EVAL was precipitated by dispersing 0.5 mL EVAL solution into 10 mL of deionized water with 1.0 mg/mL of UCNPs; then, the template was removed by washing in 10 mL deionized water 15 min (3×), separating the MC540/MNPs@MIPs/UCNPs using a magnetic field (or centrifugation at 8000 rpm, if no magnetic nanoparticles were added) for 10 min after each wash. The UCNP-embedded non-imprinted polymer-coated MC540/MNPs@NIPs/UCNPs were prepared identically, but without peptide addition. The coating of the EVAL, however, may reduce the cytotoxicity of the composite nanoparticles [22].

### 2.4. Characterization of Core/Shell UCNPs and Peptide-Imprinted Composite Nanoparticles

Wide-angle X-ray diffractograms (WAXD) were obtained with a Bruker D8 ADVANCE diffractometer, using Cu Ka radiation with a step size of 0.05° and a scanning speed of 4°/min. Transmission electron microscopy (TEM) images were obtained using a JEOL JEM1230 transmission electron microscope. Photoluminescence (PL) spectra were recorded on a Hitachi F-7000 fluorescence spectrophotometer (Japan). Scanning electron microscopy (SEM) images were obtained using a Hitachi S4800 scanning electron microscope. Fourier-transform infrared spectroscopy (FTIR) spectra were recorded using a Spectrum GX FTIR spectrometer (PerkinElmer, Inc. Waltham, MA, USA). The transmission spectra of the KBr sample pellets were collected at a resolution of 4 cm^−1^, acquiring a total of 16 scans in the wavenumber region of 400–4000 cm^−1^. Ultraviolet-visible (UV-vis) spectroscopic analysis (SP-8001, Shishin Technology Co., Ltd., Taipei, Taiwan) was performed to evaluate the binding of the PD-L1 peptide to the imprinted or non-imprinted particles. One milligram of composite nanoparticles was added into 10 mL of peptide E solution (20 μg/mL) and allowed to bind for 10 to 60 min. After magnetically removing the particles, the peptide E concentration was assayed using the absorption at 295 nm.

### 2.5. Cytotoxicity Test and Photodynamic Therapy of HepG2 Cells with MC540/MNPs@MIPs/UCNPs

**Scheme 1:**
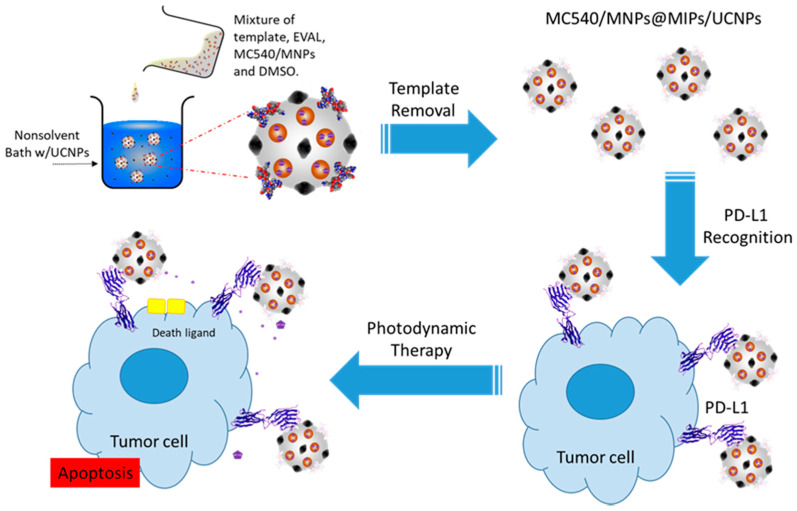
Preparation and administration of PD-L1 peptide-imprinted composite nanoparticles.

HepG2 (Human hepatoblastoma cells, BCRC #60364) cells were cultured in 90% of a 1:1 ratio mixture of Dulbecco’s modified Eagle’s medium (DMEM) and Ham’s F12 medium with 10% heat-inactivated FBS (fetal bovine serum), supplemented with 0.4 mg/mL G418 (Geneticin) at 37 °C and 5% CO_2_. For the cytotoxicity experiments, 10 μL of 10.0 × 10^4^ HepG2 cells and 190 μL of culture medium per well (1.0 × 10^4^ HepG2 cells per well) were seeded in 96-well culture plates and then incubated at 37 °C in 5% CO_2_ for 24 h. Various concentrations of nanoparticles were added to each well for another 24 h. The well plate was exposed to photodynamic light irradiation using a SDL980-LM-5000T laser diode (980 nm, 3 W/cm^2^) obtained from Shanghai Dream Lasers Technology Co., Ltd. (Shanghai, China) for 5 min. The distance between the NIR device and the cell plate was 1.5 cm. Then, 20 μL MTT (3-(4,5-dimethylthiazol-2-yl)-2,5 diphenyltetrazolium bromide, a yellow tetrazole) solution in phosphate-buffered saline (PBS) was added to each well after 24 h, and then incubated in 5% CO_2_ for 3 h at 37 °C. The solution was removed from each well. Then, 100 μL dimethyl sulfoxide (DMSO) was added to each well and incubated at 37 °C for 30 min in the dark, until the cells had lysed and the purple crystals were dissolved. The absorption intensities were measured by an ELISA reader (CLARIOstar, BMG Labtech, Offenburg, Germany) at 450 nm (I_450_), and the reference absorption (I_ref_, to account for turbidity and scattering) was obtained at 650 nm. The cellular viability (%) was then calculated from the ratio of effective absorption (I_450_-I_ref_) to controls. For the repeated assessment of cell viability, MC540/MNPs@MIPs/UCNPs and MC540/MNPs@MIPs/ UCNPs 1.0 mg/mL were added to HepG2 cells. Four hours after PDT, particles were removed from wells, 500 μL of CCK-8 (Sigma Aldrich, Kumamoto, Japan) solution was added, and the absorption intensity was measured by an ELISA reader. The preparation and administration of PD-L1 peptide-imprinted composite nanoparticle are shown in Scheme 1.

The following procedure was used for nuclear staining with DAPI (4′,6-diamidino-2-phenylindole dihydrochloride): HepG2 cells were washed 3 times using 250 μL PBS; then, 350 μL of 3.7% formaldehyde was added into each well and incubated for 15 min at room temperature. The formaldehyde solution was removed and then the cells were washed 3 times with PBS, and 250 μL of 1 μg/mL DAPI stain solution was added and incubated for 15 min at room temperature while protected from light. Finally, the DAPI stain solution was removed and cells were washed 3 times with 250 μL PBS. Imaging was performed after 5 min.

### 2.6. Gene Expression of HepG2 Cells Treated with MC540/MNPs@MIPs/UCNPs and PDT

The sequences (5′-3′) of primers for GAPDH, Caspase 8, Caspase 3, Caspase 9, Cytochrome C (CYCS), Bax, Bid, and Bcl-2 are listed in Table 1. The total RNA extraction from the HepG2 cells cultured one day after NIR irradiation was performed using the KingFisher Total RNA Kit and the KingFisher mL magnetic particle processors, both from Thermo Scientific (Vantaa, Finland). Complementary DNA was obtained following a Deoxy+ real-time 2× SYBR green RT-PCR kit (Yeastern Biotech Co., Ltd., New Taipei City, Taiwan) protocol. The real-time PCR analysis was then performed in a PikoReal real-time PCR system (Thermo Scientific, Vantaa, Finland). Relative gene expression was determined using the ΔΔCq method [23] and normalized to a reference gene (GAPDH) and to a control (HepG2).

**Table 1:** The sequence of primers used in this work, including GAPDH, Casp8, Casp3, Casp9, CYCS, Bax, Bid, and Bcl-2.

Primer	Sequence	
	Forward	Reverse
GAPDH	ACAGTTGCCATGTAGACC	TTTTTGGTTGAGCACAGG
Casp8	CTACAGGGTCATGCTCTATC	ATTTGGAGATTTCCTCTTGC
Casp3	AAAGCACTGGAATGACATC	CGCATCAATTCCACAATTTC
Casp9	CTCTACTTTCCCAGGTTTTG	TTTCACCGAAACAGCATTAG
CYCS	AAGAACAAAGGCATCATCTG	GCTATTAAGTCTGCCCTTTC
Bax	AACTGGACAGTAACATGGAG	TTGCTGGCAAAGTAGAAAAG
Bid	CTTAGCCAGAAATGGGATG	AGTCACAGCTATCTTCCAG
Bcl-2	GATTGTGGCCTTCCTTTGAG	GTTCCACAAAGGCATCC

### 2.7. Data Analysis

All experiments were carried out in triplicate and data are expressed as the mean ± standard deviation. The cellular viability and gene expression data were analyzed with Student’s *t*-test. Statistical significance was set at a *p*-value of less 0.05, with high significance at *p* < 0.001 and dramatically significant at *p* < 0.0001.

## 3. Results and Discussion

**Figure 1:**
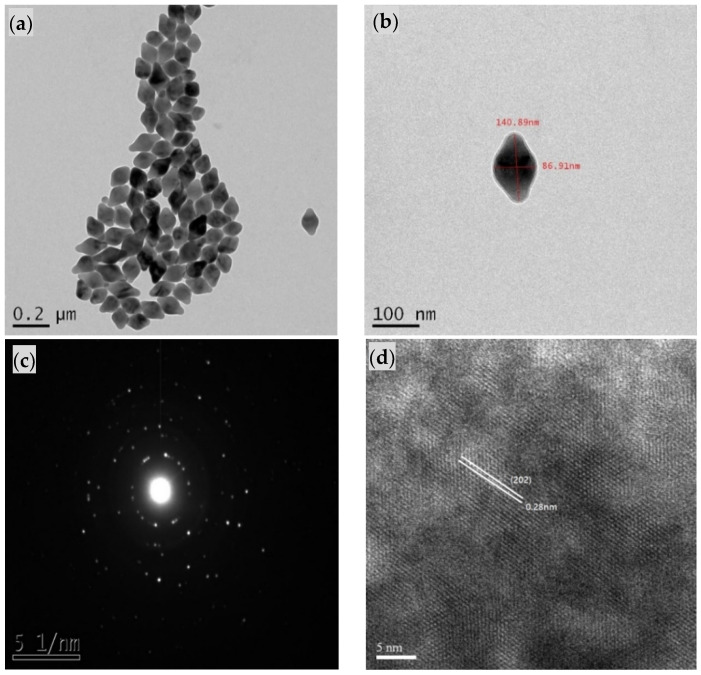
(**a**,**b**) Transmission electron microscopy (TEM) images, (**c**) electron diffraction, and (**d**) high-resolution transmission electron microscopy (HRTEM) image of LiYF_4_:Yb^3+^_0.25_/Ho^3+^_0.01_@ LiYF_4_:Yb^3+^_0.2_ UCNPs.

Figure 1 presents the morphology and crystal structures of the synthesized UCNPs. The TEM images in Figure 1a,b reveal that the nanoparticles have a tetragonal crystal structure with an octahedral morphology. The dimensions of the LiYF_4_:Yb_0.25_^3+^/Ho_0.01_^3+^@ LiYF_4_:Yb_0.20_^3+^ core/shell nanoparticles were approximately 180 ± 40 and 100 ± 15 nm along the long and short axes, respectively. In Figure 1c, high-resolution transmission electron microscopy (HRTEM) images show the well-resolved d-spacings of LiYF_4_:Yb_0.25_^3+^/Ho_0.01_^3+^@ LiYF_4_:Yb_0.20_^3+^ core/shell nanoparticles that match closely the distance between the (202) planes in lithium yttrium tetrafluoride (LiYF_4_) with a single-crystalline structure. The selected area electron diffraction patterns in Figure 1d consist of many spots that are indexed to the corresponding (hkl) planes, according to the Joint Committee on Powder Diffraction standards (JCPDs) #17-0874, indicating that the UCNPs have a single-crystal tetragonal phase structure [16].

**Figure 2:**
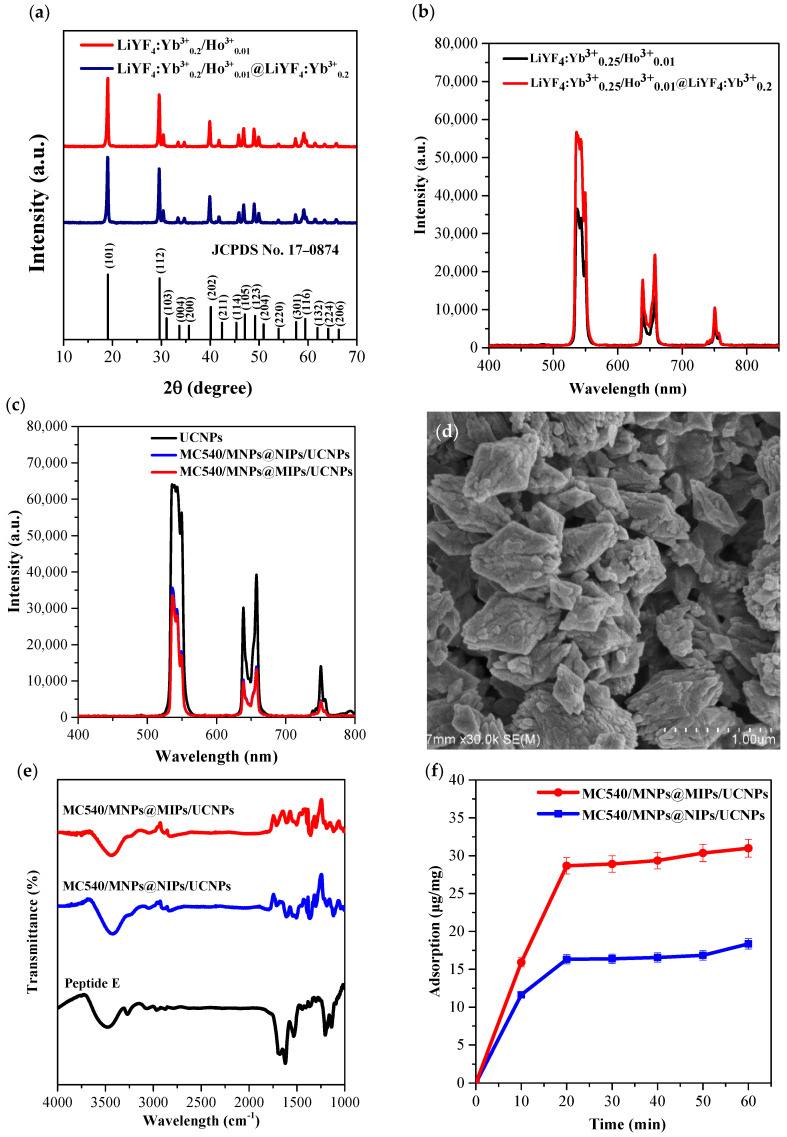
(**a**) X-ray diffraction and (**b**) photoluminescence of LiYF_4_:Yb^3+^_0.25_/Ho^3+^_0.01_ and LiYF_4_:Yb^3+^_0.25_/Ho^3+^_0.01_@ LiYF_4_:Yb^3+^_0.2_ UCNPs. (**c**) Photoluminescence of MC540-grafted MNPs at UCNPs-embedded peptide-imprinted polymer. (**d**) SEM image of the UCNP-embedded peptide-imprinted polymers. (**e**) FTIR and (**f**) binding kinetics of MC540-grafted MNPs in UCNP-embedded non- and peptide-imprinted polymers.

Figure 2 displays the characteristics of the UCNPs LiYF_4_:Yb_0.25_^3+^/Ho_0.01_^3+^@LiYF_4_:Yb_0.20_^3+^, including the XRD, photoluminescence, and adsorption of target molecules. The major peaks in the XRD diffraction patterns at 2θ values of 18, 40, 47, and 49 degrees were attributed to the (101), (112), (202), (105), and (123) planes of the tetragonal LiYF_4_ crystal. The WAXD results reveal that the relative intensities and positions of all of the diffraction peaks closely match the JCPDs #17-0874. After the LiYF_4_:Yb^3+^ shell was coated, the XRD patterns of the core/shell nanoparticles included peaks similar to those of the core nanoparticles, consistent with our previous work on LiYF_4_:Yb_0.25_^3+^/Ho_0.01_^3+^/Tm_0.01_^3+^/Er_0.01_^3+^@ LiYF_4_:Yb_0.20_^3+^ UCNPs [16]. Figure 2b,c show the photoluminescence of core, core/shell UCNPs, and MC540/MNP@MIPs/UCNPs. Interestingly, as shown in Figure 2b, the emission of UCNPs at a wavelength of 540 nm was enhanced by about 20% by coating with LiYF_4_:Yb_0.20_^3+^. The emission was quenched by about 15% when MC540 was grafted on the UCNPs. When UCNPs were embedded in MIPs or non-imprinted polymer particles (NIPs), the emission at 540 nm was further reduced (Figure 2c), perhaps because of the absorption of NIR by polymers/copolymers [24]. Figure 2d shows an SEM image of the MIPs/UCNPs; and their sizes varied from around 100 to 500 nm. FTIR measurements on the peptide, NIPs, and MIPs (Figure 2e) revealed O-H and N-H bonds around 3416 and 3351 cm^−1^ in the EVAL and peptide, respectively. The absence of the C=O, C=C, and C=N bond vibrations between 500 and 1750 cm^−1^ and of C-N around 1250 cm^−1^ showed that the template peptides had been removed from MIPs; Non-imprinted polymer (NIP) particles also lacked these vibrations, of course. Figure 2f shows the adsorption of the template (target) molecules/peptides onto the UCNPs-embedded MIPs or NIPs composite particles. The adsorption reached equilibrium at around 10 min. The adsorption capacities of the MIPs and NIPs were approximately 28.70 ± 1.08 and 16.30 ± 0.61 mg per gram, respectively.

**Figure 3:**
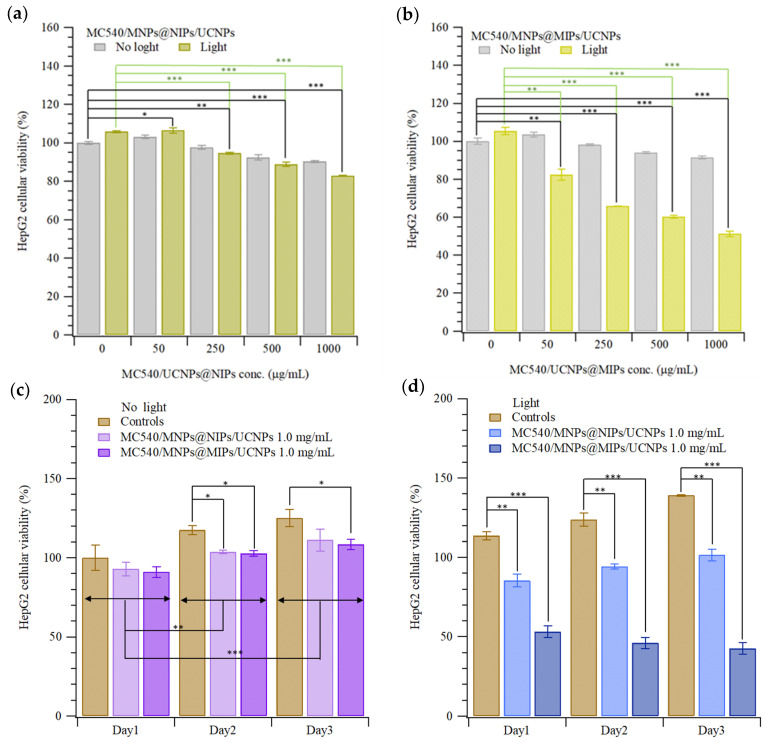
Cellular toxicity of HepG2 cells incubated with various concentrations of (**a**) NIPs and (**b**) MIPs with and without NIR irradiation (* *p* < 0.05, ** *p* < 0.001, *** *p* < 0.0001). Continuous cellular viability measurements of HepG2 cells incubated with 1.0 mg/mL of (**c**) NIPs and (**d**) PIPs/MC540 with and without irradiation (* *p* < 0.05, ** *p* < 0.001, *** *p* < 0.0001).

Figure 3a,b show the viabilities of HepG2 cells that were incubated with various concentrations of MC540/MNPs@NIPs/UCNPs (non-imprinted composite particles) and MC540/MNPs@MIPs/UCNPs (imprinted composite particles), and irradiated with 980 nm NIR for 5 min (protocols can be found in Section 2.4). Both imprinted and non-imprinted particles had only slight toxicity at the higher concentrations tested, with viabilities above 90% of that of controls. Upon irradiation with NIR, the non-imprinted particles, without a ligand for the recognition of PD-L1 on the surface of HepG2 cells, had significantly lower cytotoxicity than the imprinted particles, with viabilities about 85% of controls, even at a high particle dose. In contrast, the imprinted particles at a concentration of 1.0 mg/mL induced apoptosis in about half of the cells. The reductions in viability were highly statistically significant for MC540/MNPs@NIPs/UCNPs and MC540/MNPs@MIPs/UCNPs concentrations higher than 250 or 50 μg/mL, respectively. Since, aside from imprinting, these particles are otherwise identical, the increased efficacy of the imprinted particles must be attributed to their increased cellular binding. Figure 3c,d compare the effects of continuous administration of imprinted vs. non-imprinted composite particles on HepG2 cells. In the “continuous administration” protocol, cells were continuously incubated with particles in the culture medium, with medium replaced every day. After 1, 2, or 3 days, the cells were exposed to light for 5 min and the viability was then measured with the CCK8 kit. 

**Figure 4:**
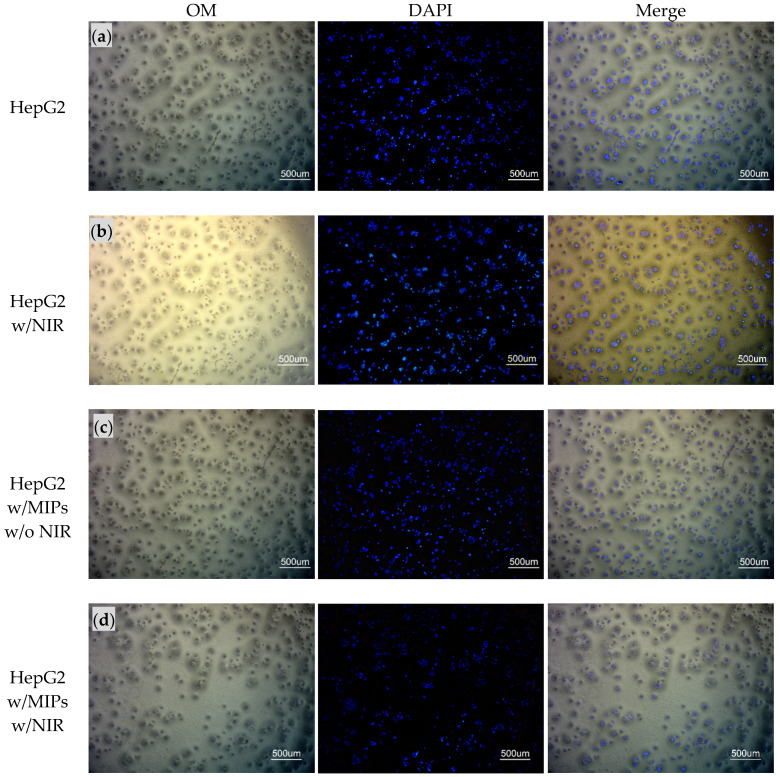
Optical, DAPI-stained, and merged images (from left to right columns) of (**a**,**b**) HepG2 cells (**c**,**d**) with MIPs under (**b**,**d**) irradiation or (**a**,**c**) not.

Figure 3c shows that the viability of cells treated with continuous administration of NPs was around 10% lower than that of the controls. Surprisingly, as shown in Figure 3d, the HepG2 cells were stimulated by the NIR and grew continuously for the first three days. The MC540/MNPs@NIPs/UCNP-treated cells had almost the same viability with and without irradiation, but compared to the irradiated controls, they grew more poorly. However, the MC540/MNPs@MIPs/UCNP-treated cells had dramatically lower viabilities than both the irradiated controls (without NPs) and the cells treated with NIPs and irradiated. These viability changes (compared with controls) were highly and dramatically significant, respectively. Figure 4 displays the cellular images of HepG2 cells that were treated with MC540/MNPs@MIPs/UCNPs. Figure 4a,b are images of cells in control experiments without and with NIR irradiation, respectively. The number of cells in Figure 4b exceeds that in Figure 4a, which agreed with the results presented in Figure 3. Figure 4c,d show HepG2 cells treated with MC540/MNPs@MIPs/UCNPs without and with NIR irradiation; many fewer cells were found compared the controls or unirradiated (particle-treated) samples. The cell counts were 703, 774, 678, and 386, from the top to the bottom of Figure 4.

**Figure 5:**
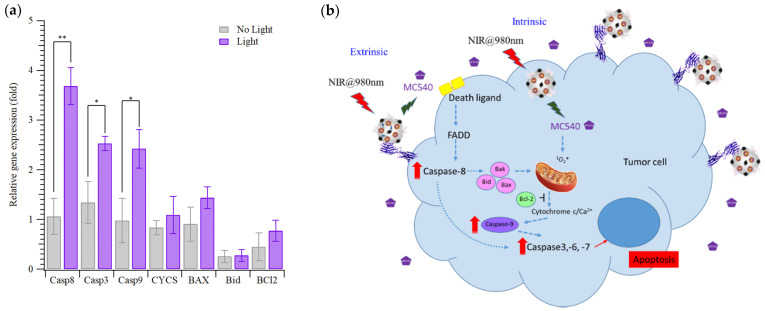
(**a**) Relative gene expression and (**b**) possible pathway of cellular apoptosis (Casp8, Casp9, Casp3, and Bax, Bid, Bcl-2, Cycs) of HepG2 cells treated with MIPs under irradiation or not (* *p* < 0.05, ** *p* < 0.001).

Finally, Figure 5 presents the gene expression levels for the intrinsic and extrinsic pathways of apoptosis. Interestingly, the major cytotoxic pathway of HepG2 cells with MC540/MNPs@MIPs/UCNPs is the extrinsic pathway, as shown in Figure 5a. The extrinsic pathway involves an increase in the expression of caspase 8 (CASP8) and then caspase 3 (CASP3), as shown in Figure 5b. The relatively higher expression of these markers on HepG2 cells treated with MC540/MNPs@MIPs/UCNPs under NIR irradiation (compared to cells without irradiation) suggests that the extrinsic apoptosis pathway has been activated.

## 4. Discussion

Precision, targeted medicine is important to reducing drug dosage and thus in minimizing side-effects in healthy cells. In this work, luminescent nanocomposite MC540/MNPs@MIPs/UCNPs were fabricated for the study of photodynamic therapy, PDT. First, green-emitting UCNPs, LiYF_4_:Yb^3+^/Ho^3+^@LiYF_4_:Yb^3+^, were synthesized by thermal decomposition. Then, the photosensitizer MC540 was loaded onto magnetic nanoparticles (MNPs), and the MNPs and UCNP were both encapsulated in MIPs synthesized to target tumors. This composite particle allows cytotoxic PDT to be employed to specifically and precisely kill tumor cells. Luminescence resonance energy transfer (LRET) from the UCNPs excites the MC540, which then catalyzes the generation of reactive oxygen species (ROS) that result in cell toxicity. The blocking the ligand of PD-L1 can also directly assist by preventing the PD-1/PD-L1 immune blockade in immunotherapy [20]. The results here show that these composite particles are effective at enhancing the efficacy of PDT and showed that the extrinsic apoptosis pathway could be activated.

We observed slight but statistically significant toxicity from the prepared composite nanoparticles, even in the absence of illumination. It may be possible to mitigate such effects using surface modification of the as-prepared composite nanoparticles. Indeed, surface modification plays a crucial role in nanomedicine, and modification with polymers, small organic molecules, or inorganic materials has been studied [25,26]. Such modifications could enhance the utility of composite MIP nanoparticles for in vivo therapy.

## Data Availability

The authors confirm that the data supporting the findings of this study are available within the article.
